# Improving the consistency of experimental swine dysentery inoculation strategies

**DOI:** 10.1186/s13567-023-01180-y

**Published:** 2023-06-16

**Authors:** Juan C. Parra-Aguirre, Roman Nosach, Champika Fernando, Janet E. Hill, John C. S. Harding

**Affiliations:** 1 Department of Large Animal Clinical Sciences, Western College of Veterinary Medicine, University of Saskatchewan, 52 Campus Dr., Saskatoon, SK S7N 5B4 Canada; 2 Department of Microbiology, Western College of Veterinary Medicine, University of Saskatchewan, 52 Campus Dr., Saskatoon, SK S7N 5B4 Canada

**Keywords:** *Brachyspira hampsonii*, *Brachyspira hyodysenteriae*, mucohemorrhagic, bloody, diarrhea, experimental, virulence, oral

## Abstract

Swine dysentery (SD) caused by pathogenic *Brachyspira* spp. is an economic challenge for the swine industry. In research settings, experimental reproduction of swine dysentery typically relies on intragastric inoculation which has shown variable success. This project aimed to improve the consistency of the experimental inoculation protocol used for swine dysentery in our laboratory. Over six experiments, we evaluated the influence of group housing in inoculated pigs using a frozen-thawed broth culture of strongly hemolytic *B. hyodysenteriae* strain D19 (Trial A), compared the relative virulence of *B. hyodysenteriae* strains D19 and G44 (Trial B), compared inoculum volumes (50 mL vs 100 mL) for G44 and *B. hampsonii* 30446 (Trial C), and performed three independent trials evaluating intragastric inoculation using different oral inoculation methods: oral feed balls (Trial D), and oral syringe bolus of 100 mL (Trial E) or 300 mL (Trial F). Intragastric inoculation with a fresh broth culture of *B. hyodysenteriae* strain G44 resulted in a shorter incubation period and a higher proportionate duration of mucohemorrhagic diarrhea (MMHD) compared to D19. Intragastric inoculation with either 50 or 100 mL of *B. hampsonii* 30446 or B. *hyodysenteriae* (G44) were statistically equivalent. Oral inoculation with 100 mL or 300 mL also yielded similar results to intragastric inoculation but was more expensive due to the additional work and supplies associated with syringe training. Our future research will use intragastric inoculation with 100 mL of a fresh broth culture containing *B. hyodysenteriae* strain G44 as it yields a high incidence of mucohaemorrhagic diarrhea with a reasonable cost.

**Supplementary Information:**

The online version contains supplementary material available at 10.1186/s13567-023-01180-y.

## Introduction

Swine dysentery (SD) is a production-limiting disease of grower or finishing pigs caused by the colonization of the large bowel by pathogenic *Brachyspira hyodysenteriae* or *B. hampsonii*, resulting in mucoid or mucohemorrhagic diarrhea (MMHD). The first description of SD was in 1921, describing the transmission of the disease in a field outbreak after the arrival of feeder hogs [1, 2] but the causal spirochete was not identified until 1972 [3]. By the mid-1990s, the prevalence of swine dysentery in the US and Canada was low, but outbreaks of bloody diarrhea in the late-2000s signalled its re-emergence [4].

Current strategies to prevent the disease include maintaining strict biosecurity measures, nutritional modifications [5–7] and the use of a limited number of experimental vaccines in some countries [8]. On the other hand, the impact of a SD outbreak is often reduced with antimicrobials, but the minimum inhibitory concentrations of some antibiotics used to treat the most pathogenic *Brachyspira* species have increased [9]. Moreover, dissemination of multi-drug resistant isolates [10] has hampered eradication programs and prompted the development of strategies to avoid the repercussions of antibiotic resistance in animal agriculture.

In experimental settings, multiple strategies have been used to induce SD in naïve pigs including direct contact with infected pigs (seeder pig model) [11], intragastric inoculation with homogenates of large intestine and its contents from affected pigs [12], or inoculation with bacterial cultures grown on solid or liquid media [13–15]. However, success of these experimental models in our lab and others has been variable in terms of inducing SD, which could be related to the additive effect of multiple animal factors including but not limited to pig age, weight, housing, diet [6, 7], genetics [16], events such as environmental and social stresses, dietary changes, or prior exposure to pathogenic *Brachyspira* spp*.* In addition, inocula-related factors such as delivery method, concentration and volume, infectious dose, strain virulence, and inoculation schedule (number and frequency of treatment) may have an impact.

The most common inoculation technique to induce SD through experimental challenge is intragastric inoculation of *B. hyodysenteriae* propagated in solid or liquid media for one or multiple consecutive days. While there are minor laboratory specific modifications, this technique has shown an incidence of MMHD ranging from 20 to 100% [11, 17–21]. In our lab, intragastric inoculation is the gold standard technique to induce SD in challenge experiments. It involves three daily inoculations following a 12–15 h fasting period, premedication with gastric acid-reducing drugs, propagation of *Brachyspira* in liquid media, and the use of sedatives prior to insertion of a feeding tube while the pig is lying in lateral recumbency. After the oral feeding tube is passed through into the stomach, the inoculum is administered, followed by a sterile volume of PBS to empty the inoculant remnant from the gastric tube. For *B. hampsonii* (genomovar* I* or *II*) incidence rates have ranged from 20 to 72%, whereas incubation period and duration of clinical signs ranged from 5 to 13 days, and 3 to 12 days, respectively [12, 13, 22]. It is clear from these studies that the results of experimental inoculation are highly variable, which is probably a reflection of the variety conditions used across the experiments.

The aim of the present study was to improve the consistency of our experimental SD inoculation protocol using broth cultures through an iterative approach including comparing the virulence of three *Brachyspira* species/strains: *B. hampsonii* genomovar II strain 30446, and *B. hyodysenteriae* strains D19 and G44. Three less-invasive oral inoculation methods (oral feedballs and oral syringe bolus of 100 or 300 mL) were also evaluated. The context of this research was to optimize an inoculation protocol that would result in near 100% incidence of MMHD with a short, consistent incubation period for use in small groups of seeder pigs (*n* = 3 to 6) in future natural transmission experiments.

## Materials and methods

### General and laboratory procedures

#### Isolates of Brachyspira hampsonii 30446, B. hyodysenteriae G44 and D19

The source of *B. hampsonii* (ST7) and *B. hyodysenteriae* G44 (ST 177) have been reported previously [11, 23, 24]. *B. hyodysenteriae* D19 is a unique field strain that does not match any reported sequence type in the PubMLST database [25] (Hill et al., unpublished). D19 originated from a fecal sample collected from a pen with MMHD in a commercial Saskatchewan farm in 2019. D19 was initially subcultured multiple times on BJ agar [26] for purification purposes prior to propagation in BHIS broth (BHI broth with 10% (v/v) FBS and 1% (w/v) glucose) for preparation of frozen aliquots for future use. Sequence typing was conducted in silico (whole-genome MLST) [25] by searching for the MLST targets established for *Brachyspira* [27] using the PubMLST database.

#### Inocula preparation and viability evaluation

The inocula were prepared as described previously [11]. Briefly, the frozen purified isolates of *B. hampsonii, B. hyodysenteriae* G44 or D19 were cultured in JBS [12] or BHIS (trial A only) broth, anaerobically incubated, and scaled up every 24-h using a 1:9 dilution of broth culture (v/v) in JBS or BHIS until the required volume for inoculation was achieved. The purity of each inoculum aliquot was assessed by PCR amplification and sequencing of the *nox* gene [28]. The concentration of *B. hyodysenteriae* D19, G44, and *B. hampsonii* in the inocula was determined by quantitative PCR [11]. For trial A that used frozen-thawed inocula, the complete volume of purified inocula was first produced, then frozen and subsequently thawed to room temperature in daily aliquots 15 min before each inoculation.

The viability of the inocula was confirmed for all trials by culturing an aliquot on BJ agar and evaluating the presence of strongly beta-hemolytic zones 48 and 96 h later. Additionally, the inocula of trials C, E and F was evaluated using a *Brachyspira* viability test. Each batch of inocula was used for inoculation only if > 95% of cells were viable.

The *Brachyspira* viability test was developed using the Bacterial Live Dead BacLight assay (Thermo Fisher Scientific, Product L7007). Briefly, equal volumes of SYTO9 (3.34 mM) and propidium iodide (20 mM) were mixed, then diluted in distilled water to achieve a 1:40 dilution. To evaluate the inoculum, 5 µL of viability stain was added to 100 µL of inoculum, vortexed, and incubated in the dark for 15 min. Following centrifugation at 1000 × *g* for 2 min, 70 µL of supernatant was discarded and the sample vortexed. Five µL of the stained inoculum were dropped between a slide and an 18 mm square coverslip. A minimum 200 *Brachyspira* cells were counted at 100 × magnification using a fluorescent microscope (Zeiss Axiovert 135) to determine the percentage of dead (red) versus live (green).

#### Inoculation

Feeders were removed from all pens for ~ 14 h prior to each inoculation and were re-introduced into the pens for about 8 h between three daily inoculations. Water was removed from the pens about 2 h before each inoculation to empty the stomach of fluid. Ninety minutes before inoculation, all pigs received an oral dose of a histamine-H2-receptor antagonist (ranitidine 0.75 mg/kg or famotidine 1 mg/kg, PO) to reduce gastric acid secretion. Additionally, on 3 consecutive days [0 to 2 days post-inoculation (dpi)], each intragastrically inoculated animal was sedated intramuscularly (IM) with xylazine (2 mg/Kg, Rompun^®^, Bayer^®^), ketamine (8 mg/Kg, Ketalean^®^, Vetoquinol^®^), and acepromazine (0.1 mg/Kg, Acevet 25^®^, Vetoquinol^®^). When sedated, an 18" French foal feeding tube was inserted while the pig was lying in lateral recumbency, and 10 mL sterile PBS (0.1 M, pH 7.0) was administered to help ensure the tube was not placed in the trachea. When correct placement was confirmed, the appropriate volume of broth culture inoculum was administered, followed by 30 mL of sterile PBS. Water was made available immediately after each inoculation.

#### Sample collection and fecal culture

Fecal samples from all pigs were collected free-fall or digitally (per rectum) scored for consistency, and plated on BJ selective agar culture plates during acclimation (4–6 times over 1–2 weeks), post-inoculation and at termination of each trial. The culture plates were evaluated based on the presence of strongly beta hemolytic zones as a proxy for fecal shedding. The presence of any zones of strong beta hemolysis was semi-quantified using the quadrant method and considered a positive result. Culture plates were interpreted by two laboratory technicians. Jugular blood was collected from each pig one day prior to the first inoculation. Sera was separated and frozen for future use.

#### DNA extraction and PCR

The DNA extraction and species-specific *B. hyodysenteriae* and *B. hampsonii* quantitative real-time PCR (qPCR) were performed as previously described in [11] from 200 mg feces and 20 mg caecal tissue. Samples were tested in duplicate and each qPCR plate included a no-template control, an extraction negative control, and a plasmid standard curve from 10^0^ to 10^7^ copies/reaction. PCR results were reported as log base 10 genome equivalents (GE) per unit (mL for broth culture). DNA was extracted from cecal tissue collected at termination from individual samples, then pooled per group, and submitted to the Prairie Diagnostic Services Inc., Saskatoon for PCR testing to detect the presence of *Lawsonia intracellularis* and *Salmonella* spp. as previously described [29, 30].

#### Pig source, housing, and diets

Animals were sourced from one of two isolated, highly biosecure commercial farms depending on the trial; Prairie Swine Centre Inc. (Saskatoon, Canada) for trials A, B, D, and Olymel (Humboldt, Canada) for trials C, E, and F. The farms were free of SD based on the historic absence of characteristic clinical signs, interviews with herd veterinarians and farm managers, review of available production records and pathology reports, and long-term absence of antibiotics in feed and water. Feces from all pigs collected during the acclimation period were culture negative for *B. hyodysenteriae* and *B. hampsonii*.

Pens had solid concrete floors with rubber mats covering 25% of the pens. Except for trial B, the lower 1/3 of each sidewall was covered with plastic arena board (trials C, E and F) or the pens were separated by a 60 cm (2 ft) space (trials A and D) to prevent cross contamination between open-sided pens. In all trials, sandbags were placed at the front of the pens to prevent cross-contamination by water and effluent on floor, and boots were rinsed between pens. Shovels were rinsed if they moved between pens, or different shovels were used per pen.

Ad libitum water and a non-medicated custom SD diet (#19277) were provided in trials A, B, D, and E. For trials C and F, a new custom *Brachyspira* diet (JH002_CFRC) was developed and fed to provide a higher concentration of insoluble fibre. However, due to an inadvertent formulation error, the limestone was omitted from this diet. Diet ingredients and nutrient specifications are described in Additional file 1.

#### Clinical assessment, termination and pathology

Fecal consistency (FCS) was scored twice daily as: 0 = normal/formed, 1 = wet cement/loose cow pie, 2 = runny/watery, 3 = mild mucoid, 3.5 = severe mucoid, 4 = mild bloody, 4.5 = severe bloody diarrhea (Additional file 2). The incidence of MMHD was based on pigs developing FCS ≥ 3 post-inoculation (diarrhea containing mucous and/or blood). For the duration of MMHD and the proportionate duration of MMHD, only the pigs that developed MMHD were included in the median calculation. Trial length (14 to 21 dpi depending on the trial) was established at a fixed time for each subgroup (Table 1). All pigs were humanely euthanized using cranial captive bolt and exsanguination at the scheduled termination day, unless euthanized earlier for humane reasons. A qualitative assessment of gross pathology was routinely performed on ileum, apex spiral colon, and cecum post-mortem, and mucosal lesions scored as: 0 = normal, 1 = mild focal hyperemic, 1.5 = mild diffuse hyperemic, 2 = moderate focal hyperemic, 2.5 = moderate diffuse hyperemic, 3 = severe diffuse hemorrhagic. A 3 × 0.5 cm piece of cecum was collected from each animal. For trials D, E and F it was subjected to *Brachyspira* culture and *nox* PCR to detect the presence of *Brachyspira* species. Positive results were confirmed by *B. hampsonii*, *B. hyodysenteriae* G44 or D19 qPCR, as appropriate for the trial.

**Table 1:** **Methodological details for six swine dysentery inoculation experiments**

Trial				Animal		Inoculum		
Id	Length (dpi)	Acclimation period (days)	Pig source (Pig age)	Diet	Housing and inoculation route	Volume Specie/strain State		Total dose (GE × 10^11^)
A	15	10	Prairie Swine Centre (6 wks)	12977	Individual IG	100 mL Bhyo D19 Frozen/thawed		10.4
					Group housed IG			
B	21	14	Prairie Swine Centre (7 wks)	12977	Individual IG	50 mL Bhyo D19 Fresh		4.92
	17					50 mL Bhyo G44 Fresh		0.51
C	19	11	Last Mountain nursery farm (7 wks)	JH002 grower	Individual IG	50 mL Bhamp 30446 Fresh		0.25
						100 mL Bhamp 30446 Fresh		0.51
	20	14				50 mL Bhyo G44 Fresh		6.87
						100 mL Bhyo G44 Fresh		13.7
D	14	9	Prairie Swine Centre (7 wks)	12977	Individual IG	100 mL Bhyo G44 Fresh		14.8
					Individual Feedballs			
E	15	14	Olymel-Krysa nursery farm (5 wks)	12977	Individual IG	100 mL Bhyo G44 Fresh		3.15
					Individual Oral-syringe 100 mL			
F	13	14	Last Mountain nursery farm (7 wks)	JH002 grower	Individual IG	100 mL Bhyo G44 Fresh		4.26
					Individual Oral-syringe 300 mL	300 mL Bhyo G44 Fresh		

dpi: days post-inoculation, wks: weeks of age, 12977/JCH002: diet names, IG: intragastric, Bhyo: *Brachyspira hyodysenteriae* (strains G44 or D19), Bhamp: *B. hampsonii* (strain 30446), GE: genomic equivalents.

Total dose = average inoculum concentration (per day) × volume administered × number of inoculation days.

### Experimental procedures

Methods specific to each trial are described below and supplemented with additional details provided in Table 1. The individual animal was considered the experimental unit for each trial.

#### Trial A. Evaluation of B. hyodysenteriae D19 using two housing strategies

The specific objective of this trial was to determine if the incidence of SD differed between individually housed versus group housed pigs inoculated with a frozen-thawed broth culture of *B. hyodysenteriae* D19. Eight healthy, 6-week old, crossbred barrow pigs were individually identified at arrival and blocked by weight into two treatment groups within one room. Four pigs were individually housed in 4ʹ × 4ʹ ft (1.21 × 1.21 m) open sided pens, and four pigs were group housed in one 8ʹ × 4ʹ ft (2.43 × 1.21 m) open sided pen. These pen sizes were consistent with those used in normal research conditions. Blinding of animal care personnel was not possible. Non-medicated custom swine dysentery diet (#19277) (Additional file 1) and ad libitum water were provided during the acclimation and post inoculation period. Each pig was intragastrically inoculated for 3 consecutive days (0–2 dpi) with 100 mL of a frozen/thawed BHIS broth culture of *B. hyodysenteriae* D19 with an average daily dose of 3.47 × 10^9^ GE/mL.

#### Trial B. Relative virulence of B. hyodysenteriae G44 versus D19

Given the unexpectedly low incidence of SD in trial A, the specific objective of trial B was to determine if *B. hyodysenteriae* D19 was as virulent as G44. Twelve healthy, 7-week old, crossbred barrow pigs, blocked by weight, were assigned to two identical rooms, each housing 6 pigs individually in 4ʹ × 4ʹ ft (1.21 × 1.21 m) open sided pens. One *B. hyodysenteriae* strain (G44 or D19) was designated per room. All personnel involved in animal handling, data collection and statistical analysis were blinded to strain used per room. Each pig was intragastrically inoculated for 3 consecutive days (0–2 dpi) with 50 mL JBS broth with an average daily dose of 3.43 × 10^8^ GE/mL for D19 and 3.28 × 10^9^ GE/mL for G44.

#### Trial C. Evaluation of inoculum volume for B. hampsonii and B. hyodysenteriae G44

In several previous experiments conducted in our laboratory, inoculation success *using B. hampsonii* was variable [11] and it was our impression that use of greater volumes of inoculum may be more effective at inducing SD. In trial C, a 2 × 2 factorial design was used to compare two volumes (50 and 100 mL) of intragastric inocula for each of two species (*B. hampsonii* and *B. hyodysenteriae* G44). Sixteen healthy, 7-week-old crossbred barrow pigs were individually allocated to eight 4ʹ × 4ʹ ft (1.21 × 1.21 m) open sided pens in one of two rooms. Each room was assigned with either *B. hampsonii or B. hyodysenteriae* G44. Beginning 0 dpi, pigs were intragastrically inoculated on 3 consecutive days using either a 100 mL (*n* = 4) or 50 mL (*n* = 4) volume of either *B. hampsonii* or G44 JBS broth culture (per room) with an average daily dose of 1.72 × 10^8^ GE/mL or 4.58 × 10^9^ GE/mL, respectively. Personnel involved in animal handling, data collection and statistical analysis during the experiment were blinded to *Brachyspira* species per room and the volume of inoculum used for each pig.

#### Trial D. Oral inoculation with 100 mL B. hyodysenteriae G44 in feedballs

Intragastric inoculation involves sedation during which animals lie in lateral recumbency and gastric motility may be reduced [31]. The objective of this trial was to evaluate an oral inoculation strategy that did not require sedation. Seven healthy, 7-week-old crossbred barrow pigs were individually housed in seven 4ʹ × 4ʹ ft (1.21 × 1.21 m) open sided pens in the same room. Pigs were blocked by weight to one of two treatment groups systematically arranged within the room with intragastric pens interposed by two oral feedball treatment pigs. During the acclimation period, pigs in the feedball group (*n* = 4) were individually trained to consume feedballs made with grower diet mixed with peanut butter (reward reinforcer) in plastic buckets fixed to the pen. At each training session, only one feedball was delivered to each pig in the feedball group. For inoculation, the feedball group received a feedball made with 100 mL G44 inoculum mixed in a small amount of feed (~ 1:10 ratio inoculum: feed). Pigs in the intragastric group (*n* = 3) were sedated and intragastrically inoculated (100 mL JBS) as previously described. Blinding was not possible for this trial. Both groups were inoculated for 3 consecutive days (0 to 2 dpi) with of an average daily dose of 4.94 × 10^9^ GE/mL.

#### Trial E. Oral inoculation with 100 mL B. hyodysenteriae G44 using a syringe

The gastric emptying time in pigs for liquid food is reported to be shorter than for solid food of equivalent nutrient density [32]. Given that feedballs in trial D were unsuccessful, the objective of trial E was to evaluate oral inoculation of non-sedated animals using a syringe. Seven healthy, 5-week-old crossbred barrow pigs, were individually housed in 4ʹ × 4ʹ ft (1.21 × 1.21 m) open sided pens. Pigs were blocked by body weight to a treatment group. During the acclimation period, the oral group (*n* = 3) were trained to consume 100 mL of JBS media until one day before inoculation. The JBS was given from a syringe with a plastic tube attached at 5 cm above shoulder height of the pig. The outside tip of the plastic tube was covered with corn syrup before each training session (reward reinforcer). Each pig in the intragastric group (*n* = 3) was sedated and intragastrically inoculated for 3 consecutive days (0 to 2 dpi) with 100 mL of a 24 h JBS broth culture of G44 with an average daily dose of 1.05 × 10^9^ GE/mL. The oral group was not sedated and inoculated for 3 consecutive days with 100 mL of the same 24 h broth culture used for the intragastric group, administered with a syringe as describe above. Blinding was not possible for this trial.

#### Trial F. Oral inoculation with 300 mL of B. hyodysenteriae G44 using a syringe

While oral inoculation in trial E showed promising results, trial F was undertaken to determine if a higher oral inoculum volume would be more effective based on the presumption that the increased volume (decreased concentration) would result in faster transit through the stomach and a greater number of viable *Brachyspira* cells surviving to colonize the colon. Ten healthy, 7-week-old crossbred barrow pigs, were individually housed in 4ʹ × 4ʹ ft (1.21 × 1.21 m) open sided pens. Pigs were blocked by body weight to a treatment group. During the acclimation period (14 days), all pigs were trained to orally consume 10 mL of BHIS media for 5 days, then 50 mL for 2 days. Four days before inoculation, the pigs to be orally inoculated with 300 mL (*n* = 5) were selected based on willingness to drink the media, and the training volume increased to 100 mL of BHIS until one day before inoculation. Training was conducted as described on trial E. Each pig from the intragastric group (*n* = 5) was sedated and intragastrically inoculated for 3 consecutive days (0 to 2 dpi) with 100 mL of a 24 h JBS broth culture of G44 with an average daily dose of 1.42 × 10^9^ GE/mL. The oral group was inoculated without sedation for 3 consecutive days with a 1:2 JBS:BHIS broth solution comprised of 100 mL of the same 24 h broth JBS culture used for the intragastric group mixed with 200 mL BHIS, administered using a syringe as describe above. The average daily dose of the intragastric and oral groups was identical, however, the oral inocula was diluted (concentration 4.73 × 10^8^ GE/mL). Blinding was not possible for this trial.

### Economic analysis (trials E and F)

An economic analysis was undertaken for trials E and F to compare the relative costs of the inoculation methods including cost of housing pre-inoculation to accommodate longer training period, additional feed used during the training period, sedatives required for the intragastric group, and culture media for inoculation and training. Previous experiences had shown that most pigs could be trained to drink from a syringe in approximately 13 days. Because of this, the acclimation period was standardized at 7 (minimum allowable) and 13 days for intragastric and oral inoculation, respectively. The cost of sedatives used in the intragastric group was based on the current purchase price and volume used. The additional cost of media required for training (100 mL and 300 mL groups) was based on the present reagent costs. Technician labour was excluded from all calculations. Cost specifications are described in Table 2 and are reported in Canadian dollars (CAD).

**Table 2:** **Partial budget comparing inoculation cost of trials E and F**

*A*) Input costs Item	Trial E-Oral 100 mL	Trial F-Oral 300 mL
Acclimation period (days)	13 (oral) 7 (IG)	
Accommodation (CAD/pig/day)	6.80	
Feed consumption (kg/day)	0.89	
Diet (CAD/kg)	1.10	
Sedatives (CAD/pig/3 days)	6.90	
Oral training media requirement (L/pig)	0.8 (JBS)	0.37 (BHIS)
IG inoculation media requirement (L/pig/3 days)	0.33	
Oral inoculation media requirement (L/pig/3 days)	0.33 (JBS)	0.33 (JBS) + 0.60 (BHIS)
JBS media (CAD/L)	59.60	
BHIS media (CAD/L)	27.50	

*B*) *Cost estimates per inoculation method*			
Item (CAD/pig)	IG 100 mL	Trial E-Oral 100 mL	Trial F-Oral 300 mL
Accommodation	47.60	88.40	88.40
Diet	6.85	12.73	12.73
Inoculation cost	19.65	19.65	36.15
Oral training		47.68	10.18
Sedatives	6.90		
Inoculation cost total	81.00	168.46	147.45

: Canadian dollars, IG: intragastric, JBS/BHIS: *Brachyspira* liquid culture media.

Cost of labor is excluded from all calculations.

### Statistical analyses

Non-parametric tests were used due to the small treatment group sizes and limitations in assessing normality. Because the trial length varied and animal condition generally decreases as MMHD (FCS ≥ 3) persists, the proportionate duration of MMHD (duration of MMHD as a percentage of trial days) was calculated as a proxy for disease severity. Group differences in the incidence of MMHD were evaluated using a 2-sided Fisher’s exact test. Because of the presence of censored data, incubation period (number days between first inoculation and first appearance of MMHD) was assessed using Kaplan Meier survival analysis for trials A to E, and Mann Whitney test for trial F. Analysis of potential group differences in the duration of MMHD (sum of days with a FCS ≥ 3), proportionate duration of MMHD used a Mann Whitney test that only included pigs that developed MMHD. Lesion scores were also analysed using a Mann Whitney test and included all animals. All variables were analysed during the post-inoculation period. The incidence of MMHD in all trials and the incubation period for trials with censored data (A to E) were analysed using the R environment for statistical computing (R version 3.5.3). Incubation period of trial F, duration of MMHD, and proportionate duration of MMHD were analysed using Stata (version 15.1). For trial C, the inoculation volume was calculated on a GE/kg basis based on the body weight of the pig at inoculation. Relationships between inoculation volume (GE/kg) and disease outcomes were evaluated using Spearman’s correlation. For all analyses, an α level of 0.05 was used to determine statistical significance.

## Results

A summary of incidence, incubation period, duration of MMHD, and proportionate duration of MMHD for all trials is presented in Table 3. During the acclimation period of all trials, no pigs were culture positive for strongly hemolytic *Brachyspira*. However, in trial E, a *Brachyspira* strain associated with intermediate beta-hemolysis was isolated in the acclimation period. It was subsequently identified as *B. murdochii* based on nox PCR and sequencing.

**Table 3:** **Summary data of incidence, incubation, duration, and duration of MMHD by trial**

Id	Group	Inoculum	Incidence^a^		Incubation (d)^b^		Duration (d)^b^		Proportionate duration %^b^	
			Value (%)	*P* value	Median (min–max)	*P* value	Median (min–max)	*P* value	Median (min–max)	*P* value
A	Individual IG	100 mL Bhyo D19	1/4 (25)	1.00	5.0	0.32	8.0	0.85	53.3	0.85
	Group housed IG		1/4 (25)		7.0		4.0		26.7	
B	Individual IG	50 mL Bhyo D19	3/6 (50)	0.54	13 (12–20)	0.01	3 (1–7)	0.24	14.3 (4.8–33.3)	0.04
		50 mL Bhyo G44	5/6 (83)		7 (2–11)		7 (1–10)		41.2 (5.9–58.8)	
C	Individual IG	50 mL Bhamp	3/4 (75)	1.00	6 (4–13)	0.53	6 (1–6)	0.43	31.6 (5.3–31.6)	0.87
		100 mL Bhamp	3/4 (75)		5 (4–7)		6 (6–7)		31.6 (31.6–36.8)	
	Individual IG	50 mL Bhyo G44	3/4 (75)	1.00	8 (3–12)	0.71	5 (4–14)	0.77	25 (20–70)	0.24
		100 mL Bhyo G44	4/4 (100)		6.5 (5–13)		10 (1–12)		50 (5–60)	
D	Individual IG	100 mL Bhyo G44	3/3 (100)	0.14	5 (4–10)	0.92	5 (4–7)	0.14	35.7 (28.6–50)	0.14
	Individual Feedballs	100 mL Bhyo G44	1/4 (25)		9		6.0		42.9	
E	Individual IG	100 mL Bhyo G44	3/3 (100)	1.00	5 (4–8)	0.28	4 (3–9)	1.00	26.7 (20–60)	1.00
	Individual Oral-syringe 100 mL		2/3 (67)		9 (7–11)		7 (5–9)		46.7 (33.3–60)	
F	Individual IG	100 mL Bhyo G44	5/5 (100)	NA	3 (2–5)	0.56	7 (6–13)	0.17	50 (42.9–92.9)	0.17
	Individual Oral-syringe 300 mL	300 mL Bhyo G44	4/4 (100)		3 (3–3)		5 (4–8)		35.7 (28.6–57.1)	

MMHD: mucohaemorrhagic diarrhea, IG: intragastric, Bhyo: *Brachyspira hyodysenteriae* (strains G44 or D19), Bhamp: *B. hampsonii* (strain 30446).

^a^Incidence values are given frequency and as percentage (in parenthesis).

^b^Incubation, duration, and proportionate duration of MMHD values are given as median of pigs that developed MMHD.

### Trial A. Evaluation of *B. hyodysenteriae* D19 using two housing strategies

**Figure 1:**
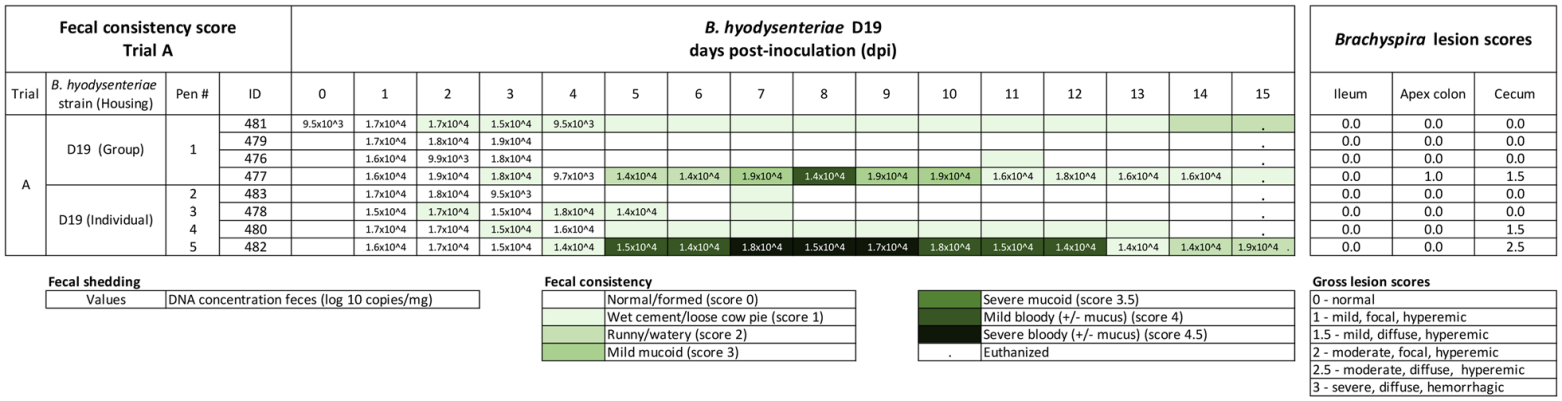
**Fecal consistency, fecal shedding and intestinal lesion scores in trial A comparing individual versus group housing. Fecal consistency by pig following inoculation with *B. hyodysenteriae*** **strain D19**. Diarrhea severity is represented by an increasing intensity of green. Fecal culture result was evaluated based on the presence ( +) or absence (−) of strong beta hemolysis. Brachyspira DNA concentration per gram feces is reported numerically. Note that only one pig per group developed mucoid or mucohemorrhagic diarrhea (MMHD).

Only 1 pig per group developed MMHD, with incubation periods of 5 and 7 days for the individual and group housed pigs, respectively. The remaining pigs from each group did not develop MMHD within 15 dpi and were terminated at the end of the study. *Brachyspira* DNA was detected by qPCR in feces of all pigs between 0 and 3 dpi indicative of inoculum passage through the gastrointestinal tract but only one pig per group showed persistent fecal shedding along with clinical signs (pig #477 and #482) (Figure 1). The duration of MMHD was 4 days in the group housed pig (#477) and 8 days in the individually housed pig (#482). Similarly, the proportionate duration of MMHD was numerically lower in the group housed pig (26.7%, 4/15) compared to the individually housed pig (53.3%, 8/15). Post-mortem evaluation showed no abnormalities in ilea and lesion scores in spiral colon apex and cecum did not differ significantly by group (Table 4). Across all pigs, mild and moderate lesions consistent of swine dysentery (score ≥ 1) were detected in spiral colon apex and cecum in 37.5% (3/8) of pigs (Figure 1), but only two pigs (#477 and 482) had MMHD during the trial. No clinical signs consistent with *Salmonella* or *Lawsonia intracellularis* were evident in ilea, spiral colon apex, or cecum. No *Salmonella* DNA was detected in cecal tissue by PCR, however, presence of *Lawsonia intracellularis* DNA was suspected (Ct = 38) in group housed pigs. In summary, the trial was inconclusive in terms of the preferred housing strategy due to the low incidence in both groups, but raised doubts regarding the use of frozen inocula.

**Table 4:** **Summary of *Brachyspira*** **lesion scores and PCR**

Group	Inoculum	Ileum^a^		Spiral colon apex^a^		Cecum^a^		PCR^b^	
		Score (min–max)	*P value*	Score (min–max)	*P* value	Score (min–max)	*P* value	*Lawsonia intracellularis*	*Salmonella*
Individual IG	100 mL *Bhyo* D19	0	NA	0	0.31	0 .8 (0–2.5)	0.40	38.0 (suspect)	ND
Group housed IG		0		0 (0–1)		0 (0–1.5)		ND	ND
Individual IG	50 mL Bhyo D19	0	NA	1.3 (0–1.5)	0.16	1.3 (0–3)	1	39.2 (suspect)	ND
	50 mL Bhyo G44	0		1.5 (0–2.5)		1.5 (0–2.5)		ND	ND
Individual IG	50 mL Bhamp	0	NA	1.5 (0–2.5)	0.34	1.5 (1.5–1.5)	0.12	ND	ND
	100 mL Bhamp	0		0.8 (0–1.5)		0.8 (0–1.5)		ND	ND
	50 mL Bhyo G44	0	NA	0.8 (0–1.5)	1	1 (0–1.5)	0.34	ND	ND
	100 mL Bhyo G44	0		0.8 (0–1.5)		0 (0–1.5)		ND	ND
Individual IG	100 mL Bhyo G44	0	NA	0 (0–1.5)	1	0 (0–1)	0.43	ND	ND
Individual Feedballs		0		0 (0–3)		0.8 (0–2.5)		ND	ND
Individual IG	100 mL Bhyo G44	0	NA	1.5 (0–2.5)	0.12	1.5 (1.5–2.5)	0.09	38.3 (suspect)	ND
Individual Oral-syringe 100 mL		0		3 (2–3)		2.5 (2.5–3)		ND	ND
Individual IG	100 mL Bhyo G44	0	NA	1.5 (0–2.5)	0.55	1.5 (0–2.5)	1	37.5 (positive)	ND
Individual Oral-syringe 300 mL	300 mL Bhyo G44	0		1.5 (0–1.5)		1.5 (0–1.5)		ND	ND

NA: not applicable, ND: non detected.

^a^Score values are given as median, refer to text for lesion scoring system.

^b^PCR values are given as Ct values.

### Trial B. Relative virulence of *B. hyodysenteriae* G44 versus D19

**Figure 2:**
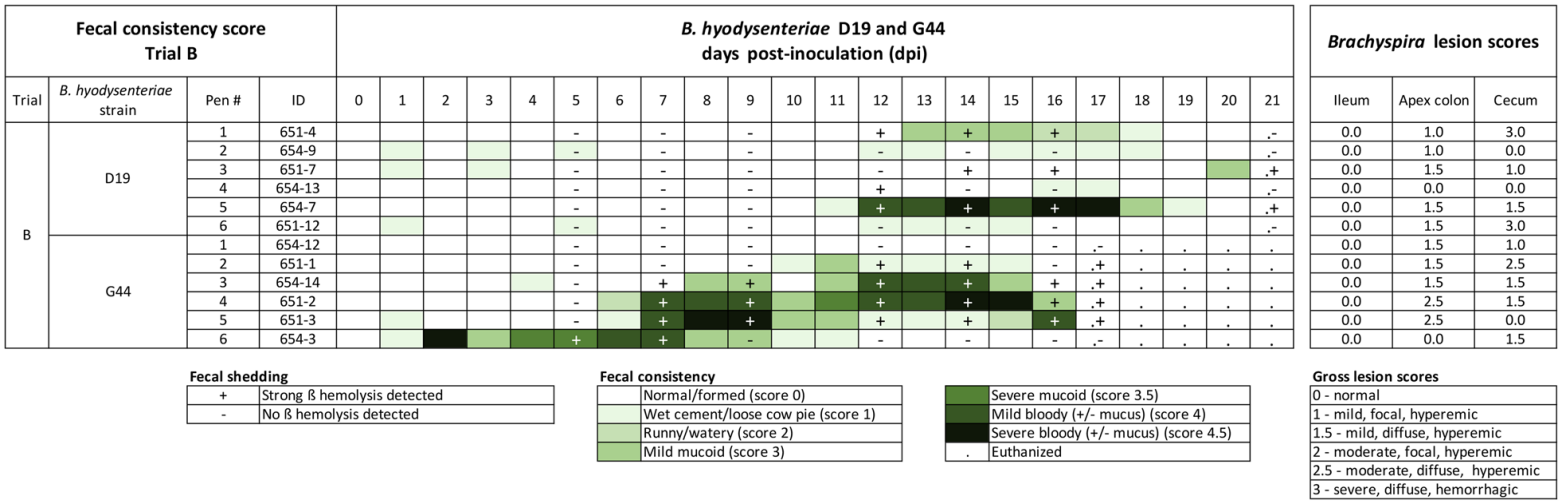
**Fecal consistency, culture and intestinal lesion scores in trial B comparing two** ***B. hyodysenteriae*** **strains.** Fecal consistency by pig following inoculation with *B. hyodysenteriae* strain D19 or G44. Diarrhea severity is represented by an increasing intensity of green. Fecal culture result was evaluated based on the presence ( +) or absence (−) of strong beta hemolysis. Note the greater incidence and duration of diarrhea when inoculated with the culture broth of *B. hyodysenteriae* G44 compared to inoculation with *B. hyodysenteriae* D19.

Inoculation with G44 resulted in a numerically higher incidence of MMHD (5/6) compared to D19 (3/6), however, this difference was not statistically significant. Pigs inoculated with G44 had a shorter (*P* = 0.01) incubation period (median 7, range 2–11 days) than pigs inoculated with D19 (median 13, range 12–20 days). No significant differences were found in duration of MMHD (*P* = 0.24) between strains, however, the proportionate duration of MMHD was higher (*P* = 0.04) in pigs inoculated with G44 (median 7/17, 41.2%, range 5.9–58.8%) compared to D19 (median 3/21 14.3%, range 4.8–33.3%) (Figure 2) (Table 3). On post-mortem, no abnormalities in ilea were observed, and lesion scores of spiral colon apex and cecum did not differ by group (Table 4). Across all pigs, mild to severe lesions consistent of swine dysentery (score ≥ 1) were detected in spiral colon apex and cecum in 91.6% (11/12) of pigs (Figure 2). Of the 11 pigs with lesions, eight showed at least one day with FCS ≥ 3. The remaining three pigs (#654-9, 651-12, 654-12) had mild to severe lesions but did not have clinical signs of SD or fecal shedding during trial. No clinical signs of *Salmonella* or *Lawsonia intracellularis* were evident in ileum, spiral colon apex, or cecum. PCR analysis of cecal tissue did not detect of *Salmonella* DNA, however, *Lawsonia intracellularis* DNA was suspected (Ct = 39.2) in pigs inoculated with D19. In summary, *B. hyodysenteriae* G44 was more virulent than D19.

### Trial C. Evaluation of inoculum volume for *B. hampsonii* and *B. hyodysenteriae* G44

**Figure 3:**
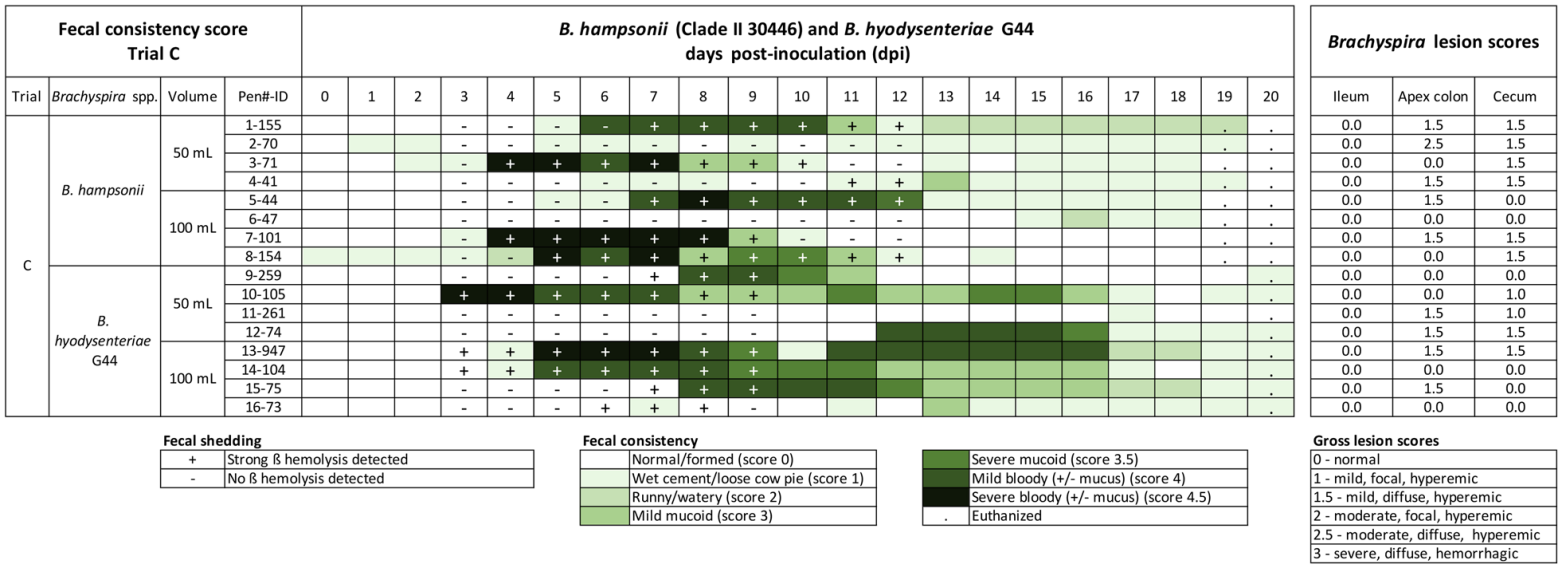
**Fecal consistency, culture and intestinal lesion scores in trial C comparing relative virulence of *B. hyodysenteriae*** **G44 and** ***B. hampsonii***. Fecal consistency scores following intragastric inoculation with *B. hampsonii* strain 30446 or *B. hyodysenteriae* strain G44 using either 100 or 50 mL inoculation volume. Diarrhea severity is represented by an increasing intensity of green. Fecal culture result was evaluated based on the presence ( +) or absence (−) of strong beta hemolysis. No differences in SD outcome were observed among group.

There were no significant statistical differences in incidence, incubation period, duration or proportionate duration of MMHD by volume of inoculum for either *B. hampsonii* or *B. hyodysenteriae* (Table 3) or by species (*P* > 0.4 for all). The incidence of MMHD in all groups was 75% or greater. The median proportionate duration of MMHD was 25% (5/20, range 20–70%) and 50% (10/20, range 5–60%) following inoculation with 50 or 100 mL of *B. hyodysenteriae* G44, respectively. The median proportionate duration of MMHD was 31.6% (6/19) for both 50 or 100 mL of *B. hampsonii*, with a slight difference in the range (5.3–31.6% for 50 mL, 31.6–36.8% for 100 mL) (Figure 3). Inoculation concentration (GE/kg) ranged from 9.52 × 10^9^ to 2.32 × 10^10^ for *B. hyodysenteriae*, and 3.61 × 10^8^ to 98.96 × 10^8^ for *B. hampsonii*, but was not correlated with incubation period, average FCS, duration, or proportionate duration (*P* > 0.1 for all) in either *Brachyspira* spp.. There were no ileal lesions observed and no statistical group differences in lesion scores in colon or cecum (Table 4). Across all pigs, mild and moderate lesions consistent of swine dysentery (score ≥ 1) were detected in colon apex and cecum in 75% (12/16) of pigs (Figure 3). Of those with lesions, all showed MMHD at least one day except two animals (#2-70 and 11-261) that were both repeatedly culture negative. No evidence of *Lawsonia intracellularis* or *Salmonella* were observed based on gross pathology or PCR. In summary, the inoculation volumes used had no effect on outcome in either species, however, only G44 at 100 mL resulted in 100% disease incidence.

### Trial D. Oral inoculation with 100 mL *B. hyodysenteriae* G44 in feedballs

**Figure 4:**
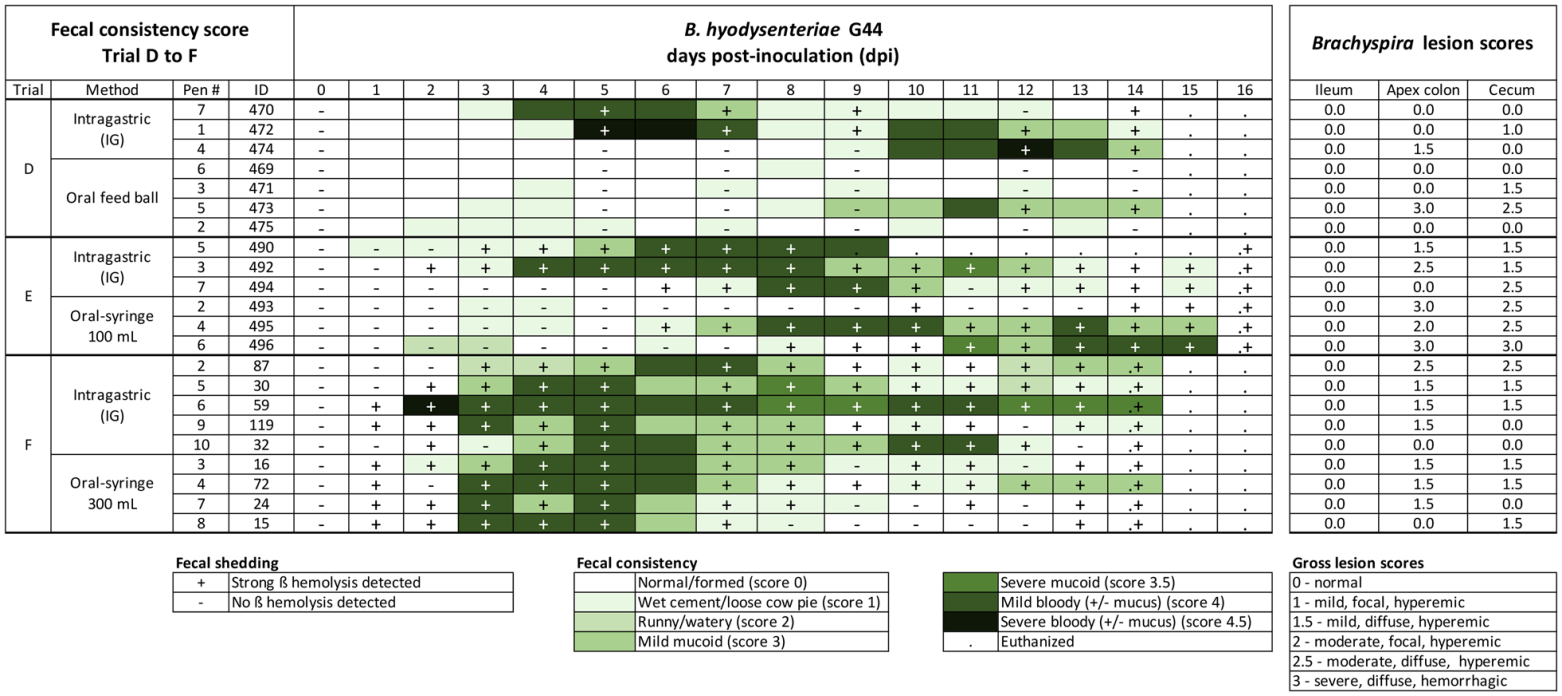
**Fecal consistency, culture and intestinal lesion scores in trials evaluating inoculation using feedballs (trial D), oral 100 mL inoculation (trial E), and oral 300 mL inoculation (trial F).** Fecal consistency scores following intragastric versus oral or feedball inoculation with *B. hyodysenteriae* strain G44. Diarrhea severity is represented by an increasing intensity of green. Fecal culture result was evaluated based on the presence ( +) or absence (−) of strong beta hemolysis. Note the greater incidence and duration of diarrhea using the intragastric inoculation compared to feedballs in trial D.

Inoculation using feedballs resulted in 1/4 (25%) pigs developing MMHD (Figure 4) with an incubation time of 9 days, duration of MMHD of 6 days, and a median proportionate duration of MMHD of 42.9% (6/14). Feces from this animal was culture positive on 12 and 14 dpi. The remaining three pigs in this group did not develop MMHD or a culture positive result by 14 dpi. In contrast, all three pigs challenged intragastrically developed MMHD with a median incubation of 5 days (range 4–10) and median duration of MMHD of 5 days (range 4–7). The median proportionate duration of MMHD was 35.7% (5/14, range 28.6–50%). The incidence of MMHD, incubation, duration and proportionate duration of MMHD percentage did not differ significantly between the inoculation strategies (Table 3). *Brachyspira* fecal shedding was confirmed in all pigs of the intragastric group. At termination, *Brachyspira* was isolated from caecal tissue by culture in 1/4 (25%) pigs in the feedball group and in all the pigs (3/3) in the intragastric group (Figure 4). All ilea were grossly normal and no significant differences were found in lesion scores in colon apex and cecum (Table 4). Across all pigs, mild and severe lesions consistent of swine dysentery (score ≥ 1) were detected in colon apex and cecum in 57.1% (4/7) of pigs (Figure 4). From those with lesions, all animals showed at least five days with FCS ≥ 3 except one pig (#471) with no MMHD during the trial. By contrast, one pig (#470) had 4 days with MMHD and *Brachyspira* shedding but did not show gross lesions on necropsy. No *Lawsonia intracellularis* or *Salmonella* were detected by PCR. In summary, in spite of no significant differences between group, the 100% incidence of MMHD and shedding in the intragastric group suggests it is more consistent than feedballs.

### Trial E. Oral inoculation with 100 mL *B. hyodysenteriae* G44 using a syringe

One pig from the oral group (#491) was feverish and reluctant to stand on -2 dpi from a non-SD related condition. Data from this pig was excluded from the statistical analysis. The oral inoculation of 100 mL of *B. hyodysenteriae* G44 using a syringe resulted in 2/3 (67%) pigs developing MMHD, with a median incubation period of 9 days (range 7–11). One pig (#493) did not develop MMHD, but did shed bacteria in feces on 10, 14 and 15 dpi. The median duration of MMHD was 7 days (range 5–9), and median proportionate duration of MMHD was 46.7% (7/15, range 33.3–60%). Following intragastric inoculation, 3/3 (100%) pigs developed MMHD, with a median incubation time of 5 days (range 4–8) and median duration of MMHD of 4 days (range 3–9). Two intragastrically inoculated pigs (#492 and #494) were still showing signs of MMHD on 15 dpi (trial termination) (Figure 4). The median proportionate duration of MMHD was 26.7% (4/15, range 20–60%). *Brachyspira* shedding in feces was confirmed in all intragastrically inoculated pigs (6/6 100%) and *B. hyodysenteriae* G44 was isolated from intestinal tissue collected at termination from all pigs regardless of group. All ilea were grossly normal. Lesion scores in cecum trended higher (*P* = 0.09) in the orally inoculated pigs (Table 4), and were numerically higher in colon apex (*P* = 0.12) compared to the intragastrically inoculated group. All animals had mild to severe lesions consistent with swine dysentery (score ≥ 1) and showed at least three days with FCS ≥ 3, except one pig (#493) that shed *Brachyspira* for 4 days but did not develop MMHD. No lesions consistent with *Salmonella* or *Lawsonia intracellularis* were observed and no *Salmonella* DNA was detected. However, *Lawsonia intracellularis* DNA was suspected (Ct value = 38.3) in pigs orally inoculated with 100 mL. In summary, there were no group differences in the incidence, incubation period, duration or proportionate duration of MMHD (Table 3) between group, however, the cost of inoculation was greater for the oral strategy (168.46 CAD) compared to the intragastric strategy (81 CAD) by 87.45 CAD per pig (Table 2).

### Trial F. Oral inoculation with 300 mL of *B. hyodysenteriae* G44 using a syringe

All ten pigs developed MMHD, however, one pig from the oral group (#NN) did not consume the entire dose (180/300 mL) of inoculum on each inoculation day. Data from this pig was excluded from the statistical analysis and pathology evaluation. All pigs orally inoculated with 300 mL had a median incubation period of 3 days (no range), median duration of MMHD of 5 days (range 4–8), and median proportionate duration of MMHD of 35.7% (5/14, range 28.6–57.1%). *Brachyspira* fecal shedding was confirmed in 4/4 (100%) pigs, and *B. hyodysenteriae* G44 was isolated from intestinal tissue collected at termination in all pigs. In the intragastric inoculation group, the median incubation period was 3 days (range 2–5), median duration of MMHD was 7 days (range 6–13), and median proportionate duration of MMHD of 50.0% (7/14, range 42.9–92.9%). Fecal shedding of *B. hyodysenteriae* G44 was confirmed in all pigs, as well as in intestinal tissues collected at necropsy (Figure 4). No group differences in the incidence, incubation period, duration of clinical signs, or proportionate MMHD duration (Table 3). Post-mortem evaluation showed no abnormalities in ilea. No statistically significant group differences were seen in lesion scores of spiral colon apex and cecum (Table 4). Across all pigs, mild and moderate lesions consistent of swine dysentery (score ≥ 1) were detected in colon apex and cecum in 88.8% (8/9) of pigs (Figure 4). All eight animals showed at least four days with FCS ≥ 3 during trial. Lesions consistent with *Salmonella* or *Lawsonia intracellularis* infection were not observed, and *Salmonella* DNA was not detected in cecal tissue. A low level of *Lawsonia intracellularis* DNA was detected (Ct value = 37.5) in the intragastrically inoculated group. In summary, both techniques were very successful but the cost of inoculation per pig was 66.45 CAD greater for the 300 mL oral inoculation group (147.45 CAD/pig) compared to the intragastric strategy (81 CAD/pig) (Table 2).

## Discussion

The development of a consistent natural transmission model for SD relies on successful seeder pig inoculation that results in mucohemorrhagic diarrhea with a consistent, short incubation period, high incidence, and long duration with persistent fecal shedding. The findings from this study underscore that the *Brachyspira* strain, inoculation method, and state of the inoculum (frozen or fresh) may influence the consistency and reliability of experimental SD challenge models using broth culture inoculum.

Multiple *B. hyodysenteriae* genotypes have been reported with important strain variation worldwide [23]. Emerging *Brachyspira* species and strains require the development of fast and cost-effective diagnostic tools for identification, as well as techniques to evaluate changes in biological properties and virulence properties. The intensity of the beta-hemolysis observed on blood agar plates has been used to infer virulence potential. While normally strongly hemolytic, there are reports of low virulence, weakly hemolytic *B. hyodysenteriae* strains [33]. Currently, techniques like the MLST have been used in an international effort to identify and track the spread of new clonal groups of *B. hyodysenteriae* [27]. In the present study, a Canadian *B. hyodysenteriae* strain named “D19” was isolated from a clinical case and used in two challenge trials. Unexpectedly, the intragastric inoculation of a frozen-thawed culture broth of *B. hyodysenteriae* D19 resulted in only one pig developing MMHD using either the individual or group housing strategy. Other researchers evaluating the efficiency of intragastric inoculation using frozen-thawed *B. hampsonii* inocula also reported an inconsistent frequency, duration, and onset of disease when compared to fresh inocula [34]. While this suggests that frozen inocula may be less successful, the result must be interpreted with caution because side-by-side experiments comparing fresh and frozen-thawed inocula have not been rigorously undertaken.

Intragastric inoculation of fresh broth culture was used to evaluate the relative virulence of three *Brachyspira* species/strains (*B. hampsonii, B. hyodysenteriae* G44, and *B. hyodysenteriae* D19). In trial B, *B. hyodysenteriae* G44 was observed to be more virulent than *B. hyodysenteriae* D19, as evidenced by the shorter incubation period and a higher proportionate duration of MMHD, along with a numerically (non-significant) higher incidence of MMHD. Although genes related to virulence have shown to be well conserved across *B. hyodysenteriae* isolates, gene variation may contribute to an increase in the pathogenicity of some *B. hyodysenteriae* strains [35], including but not limited to virulence and/or virulence lifestyle genes related to aero-tolerance, hemolysis, iron metabolism, and outer membrane proteins [36]. In terms of future research, it would be useful to compare gene expression patterns of each of the evaluated strains.

*B. hampsonii* (clade/genomovar II) produces similar, if not indistinguishable, lesions compared to those resulting from infection with *B. hyodysenteriae* 204 [15]. Moreover, *B. hampsonii* and *B. hyodysenteriae* G44 have shown similarities in the mechanisms that lead to malabsorptive diarrhea [37]. In trial C, there were no statistically differences in incidence, incubation period or duration between the species (*B. hyodysenteriae* G44 or *B. hampsonii*) or between inoculation volume (50 vs. 100 mL) administered intragastrically indicating the similar virulence of both species. Moreover, the concentration of intragastric inocula calculated on a GE/kg basis was not associated with disease outcome. This result suggest that inoculation success is not dose dependent provided a theoretical minimum infective dose is achieved and sufficient organisms survive gastric acid exposure. While the present experiments were not designed to establish a minimum infective dose, it is noteworthy that all pigs G44 inoculated intragastrically with > 2.37 × 10^11^ GE total dose over three days developed MMHD. In a previous study by our lab [11], an unsuccessful attempt to induce SD using 50 mL intragastric *B. hampsonii* inoculation (1.05 × 10^11^ GE total dose over 3 days) was followed by the full induction of SD in all pigs after *B. hyodysenteriae* G44 reinoculation with 2.5 × 10^11^ GE total dose over three days. Whether these inoculation doses truly reflect of a minimum infectious dose is unknown but may be a starting point for further investigation. Although trial results from different labs need to be compared with extreme caution due to differences in the *Brachyspira* strain used and experimental protocols, research groups experiencing low or variable inoculation success should consider evaluating minimum infectious dose specific to the experimental conditions used in their laboratories. Examples of previous published research help to highlight this point and how challenging *Brachyspira* inoculation can be. For instance, intragastric inoculation of 40 mL of a strongly hemolytic strain of *B. hyodysenteriae* (strain B204) broth containing 1 × 10^8^ colony forming units (CFU)/mL for 3 days (1.2 × 10^10^ CFU total dose) resulted in 55.5% (5/9) SD incidence (Trial 2 of [20]), whereas intragastric inoculation of 50 mL (1 × 10^9^ CFU/mL) of a Danish field isolate of *B. hyodysenteriae* broth following a similar methodology (1.5 × 10^11^ CFU total dose) resulted in only 20.8% (10/48) incidence of SD by another laboratory [21]. This variability in incidence highlights the need to establish minimum infective doses for the *Brachyspira* species/strains and experimental conditions specific to each laboratory, and the importance of full disclosing all methodological details in studies to help decipher differences among *B. hyodysenteriae* strains, environmental factors, and host related factors like genetics and microbiota. To improve the consistency of inoculation, the *Brachyspira* research community may benefit if a limited number of geographically relevant reference strains were agreed upon for future research.

Previous studies have shown success when inoculating by feed. All non-vaccinated pigs (5/5) inoculated using feed mixed with *B. hyodysenteriae* B204 broth on two successive days developed MMHD [38], whereas the inoculation with agar from plates cultured with a mixture of strongly hemolytic *B. hyodysenteriae* strains chopped in feed during four successive days resulted in 70.83% (17/24) incidence of SD [14]. In trial D of the current study, only one animal inoculated with the oral feed ball method (1.48 × 10^12^ GE total dose over 3 days) developed MMHD, whereas 3/3 intragastrically inoculated pigs did. Although group differences in this trial were not statistically different, the lack of consistency with the feedball method at our laboratory indicates there is no advantage of employing this technique at this time.

Oral delivery of liquid inoculum offers numerous advantages over intragastric inoculation if it is as effective. In one previous study, the oral inoculation of a strongly hemolytic strain of *B. hyodysenteriae* (strain B204) for two consecutive days resulted in 100% (5/5) incidence of SD, however, too few methodological details were provided to verify the inoculation strategy [39]. Previous experiments have shown that inoculation for two consecutive days using a mixed inoculation strategy of oral (pure cultures) or intragastric inoculation (broth culture) followed by feed with inocula, resulted in 75% (52/68) incidence and no clinical disease for 25 strongly and 13 weakly hemolytic strains, respectively [40]. Other studies have shown less favourable results. For example, the oral inoculation of 40 mL of a strongly hemolytic strain of *B. hyodysenteriae* (strain 8dII) broth administered for three consecutive days induced SD in 33% (2/6) of pigs (trial 1 of [20]). In trials E and F of the present research, oral inoculation of pigs on three consecutive days with 100 mL (3.15 × 10^11^ GE total dose over 3 days) or 300 mL (4.26 × 10^11^ GE total dose over 3 days) of *B. hyodysenteriae* strain G44 broth culture resulted in MMHD incidence of 67% (2/3) and 100% (10/10), respectively. Although oral and intragastric inoculation resulted in equivalent outcomes in these trials, the cost of oral inoculation was substantially more than intragastric inoculation. These results highlight that oral inoculation can be an effective method to induce MMHD in a susceptible population without the use of sedatives or skilled workers manually restraining the pigs, and the value of performing an economic analysis to inform choice of inoculation strategy.

The dietary composition has been suggested to be an important risk factor for the development of SD. However, the protective or mitigating effects of carbohydrates and fiber types against SD infection have been conflicting in several studies. Pigs fed with a highly digestible diet using cooked rice or with high levels of fiber resistant to complete enzymatic degradation (e.g. inulin at 80 g/kg) but not lupin offered protection from the disease after *B. hyodysenteriae* challenge [41, 42]. Recent studies evaluating different levels of soluble or insoluble dietary fiber have also showed variable results. On one hand, fiber concentration (high vs. low) and type (soluble vs. insoluble) did not effect fecal score in a Danish study, however, this result should be taken with caution due to the low incidence of MMHD across all groups [21]. On the other hand, several USA studies indicate high concentration of insoluble fiber in the diet promotes expression of SD. For example, the addition of 30% distillers’ dried grains with solubles (DDGS) shortens the incubation period of clinical signs and shedding after challenge with strongly hemolytic *B. hampsonii* (strain EB107) or *B. hyodysenteriae* (strain B204) [43]. DDGS are co-products from the biofuel and milling industries used in swine diets to reduce the cost of feeding. Unfortunately, most of these co-products are lowlily fermentable, have low nutritive value for pigs, and have a high fiber content. Recent studies have demonstrated that replacing insoluble fibre (provided as DDGS) with highly fermentable fiber and resistant starch (provided as sugar beet pulp and resistant potato starch) delayed the onset and reduced the incidence of SD following the challenge with *B. hyodysenteriae* [6, 7]. In the present study, pigs were fed with a commercial grower diet (#12977), or a custom diet (JH002) manufactured to increase the susceptibility to SD infection by increasing the insoluble fibre content using ingredients such as DDGS, wheat bran, and canola meal, as well as using highly digestible carbohydrates such as high-quality wheat to minimize the colonic fermentation.

Infection with *Lawsonia intracellularis* is characterized by proliferation of immature crypt cells in jejunum and ileum resulting in proliferative hemorrhagic enteropathy or intestinal adenomatosis, the acute and chronic presentations of the disease, respectively. A recent study found correlation between the presence of *B. hyodysenteriae* and *Lawsonia intracellularis* in European farms*.* Additionally, mixed infection seems to be frequent in countries including Germany, Denmark and the United Kingdom [44]. In the present trials, 4/14 pooled cecal samples were PCR positive at termination with high Ct values (very low DNA levels). Positive samples were inconsistent across treatment groups, unrelated to pig source, only in a single group in any trial, and not associated with characteristic gross lesions of *Lawsonia* infection. For these reasons, *Lawsonia* infection is unlikely influencing the incidence or severity of SD in the present trials. Infection with *Salmonella* is associated with thickened jejunum and colon with necrotic debris on the mucosal surface. Although *Salmonella* can coexist with *Lawsonia intracellularis* and *Brachyspira* spp., the frequency of mixed infections involving *B. hyodysenteriae* and *Salmonella* is not common as evidenced in Polish pig herds [45]*.* In the present study, no *Salmonella* DNA was detected. The *Salmonella* PCR was designed for use on feces following enrichment, but this step was omitted because DNA extracted from cecal tissue was used instead. That said, there was no evidence of infection contributing to *Brachyspira* severity. Gross lesions at termination were indicative of *B. hyodysenteriae* and *B. hampsonii* infection (score ≥ 1) with no lesions indicative of other disease processes. However, individual lesion scores varied widely from normal to severe which is likely related to the clinical state at termination (no clinical disease, recovered or active MMHD) with higher lesion scores likely reflecting longer incubation periods, more severe infection and/or longer duration.

The omission of limestone from the JH002 diet fed in trials C and F was unintentional. This diet was developed hastily to overcome supply issues when the previous diet (#12977) could not be supplied. While the omission of limestone certainly affected the dietary calcium level, calcium: phosphorus ratio, and acid binding capacity, what effect it had on SD susceptibility is unknown. In theory, pigs consuming the JH002 diet would have a decreased acid binding capacity and more acidic contents that would have adversely affected *Brachyspira* survival in the stomach and upper intestines. However, based on the high incidence of MMHD in trials C and F, this inadvertent issue is not likely an influential factor.

These trials were designed and implemented using an iterative strategy, with each one building on previous results. The trials were undertaken over a 24- month period during which there were changes in pig source, feed supplier and diet formulation. Some experimental procedures such as the *Brachyspira* viability test were implemented for later trials, while others evolved or were adapted to accommodate improvements and/or resource limitations. Despite these limitations and variations in the experimental protocols, this series of trials was useful for our laboratory to overcome inoculation challenges and develop best practices. The small sample sizes used were a reflection of the limited resources available, and more importantly, the overarching context of the research. Our natural transmission experiments [11] use a small group of inoculated seeder pigs to naturally transmit *B. hyodysenteriae* or *B. hampsonii* via fecal shedding to a larger group of naïve contact pigs using a 1:2 or 1:3 seeder:contact ratio. A near 100% incidence of MMHD and a short, consistent incubation period is required following seeder pig inoculation to ensure the exposure period (co-mingling of seeder and contact pigs) is consistent across multiple pens. The small group sizes used in these trials reflect that context, and the results informative despite the lack of statistical differences reported in most trials herein. Outliers and non-responder seeder pigs are very problematic in a natural transmission experiment because there is limited capacity to inoculate extra seeder pigs to compensate for a low incidence of MMHS, or pigs with outlier incubation periods or short duration of clinical signs and shedding. For our purposes, an inoculation protocol resulting in a consistently high incidence (near 100%) in small groups of inoculated pigs is imperative. Moving forward, further work could be done to determine the minimum infective dose for relevant *Brachyspira* strains, modifying inoculation techniques to accommodate larger pigs, and assessing the effect of host genetics and season on inoculation consistency. For these research experiments, larger group sizes would be warranted. However, given the challenges and historic inconsistency of inoculation faced by researchers around the world, best practices for inoculation will need to be laboratory specific based on the experimental conditions, expertise and local resources available.

In conclusion, under the conditions of the trials reported herein, intragastric inoculation with *B. hyodysenteriae* G44 in individually housed grower pigs yielded a more consistent model compared with *B. hyodysenteriae* D19. No significant differences were found when comparing inoculation with *B. hyodysenteriae* G44 to equivalent challenge with *B. hampsonii* 30446. Furthermore, the intragastric inoculation of 100 mL of fresh broth culture containing ~ 10^8^ genomic equivalents (GE)/mL *B. hyodysenteriae* G44 yielded the most consistent and lowest cost model for the experimental reproduction of swine dysentery in a susceptible grower pig population under the experimental conditions used in this research.

## Supplementary Information

**Additional file 1: Custom Brachyspira #12977 and JH002 diet ingredient and nutrient specs.**

**Additional file 2: Fecal consistency score.**

**Additional file 3: Arrive 10 check list.**

**Additional file 4: Humane Intervention Point checklist.**

**Additional file 5: Raw data for all trials.**

## Data Availability

All raw data related to the trials published herein are included in Figures 1, 2, 3, 4 and Additional file 5.
