# Supplementary material for: Improving the consistency of experimental swine dysentery inoculation strategies
Source: Vet Res. 2023 Jun 16;54:49. doi: 10.1186/s13567-023-01180-y (PMC10276399; doi:10.1186/s13567-023-01180-y)
Supplement: Supplementary file 1 — Additional file 1: Custom Brachyspira #12977 and JH002 diet ingredient and nutrient specs. [file 13567_2023_1180_MOESM1_ESM.pdf]

Additional file 1. Custom Brachyspira diet ingredients and nutrient specs

Custom 12977 diet

Ingredient	%	Nutrient	%
Barley	25	Crude protein	16
Wheat	30	Crude Fiber	5.6 (8% Max)
Corn DDGS	25	Crude Fat	3.6 (2% Min)
Soybean meal	10	Digestible Energy	3120 Kcal
Oat hulls	7.5	Calcium (total)	0.6
Calcium carbonate	1.05	Phosphorus (total)	0.5
Canola oil	0.5	Sodium	0.2
Salt (NaCl)	0.37	Lysine (total)	0.84
Lysine	0.25	Methionine (total)	0.3
Phytase	0.022	Threonine(total)	0.62
Micro	0.31	Tryptophan (total)	0.19
**Total**	**100**	Met + Cys (total)	0.62
		Vit A (IU/kg)	5200
		Vit D (IU/kg)	680
		Vit E (IU/kg)	25
		Copper (ppm)	6
		Zinc (ppm)	100

Custom JCH02_CRFC diet

Ingredient	%	Nutrient	%
Barley	0	Crude protein	21.1
Wheat (hard, spring)	42.24	Crude Fiber	5.9
Corn DDGS	25	Crude Fat (ether extract)	6.42
Soybean meal (50%)	4.17	Digestible Energy (NE)	2,400 Kcal
Canola meal (40%)	16.46	Calcium (total)**	0.21
Wheat bran	7.5	Phosphorus (total)	0.61
Calcium carbonate**	0	Sodium	0.28
Canola oil	3.5	Lysine (SID)	0.95
Salt (NaCl)	0.3	Methionine (SID)	0.32
Lysine	0.4	Threonine (SID)	0.59
Phytase	0.02	Tryptophan (SID)	0.19
Micro	0.4	Met + Cys (SID)	0.72
**Total**	**100**	Acid detergent fiber	7.24
		Neutral detergent fiber	22.85
		Cellulose	9.85
		Lignin	2.43
		Copper (ppm)	37.6
		Zinc (ppm)	179

* Manufactured with 10/64 screen with a target particle size of 700 microns
** inadvertent error with limestone omitted
NE=net energy, SID=standardized ileal digestibility
