# Supplementary material for: Improving the consistency of experimental swine dysentery inoculation strategies
Source: Vet Res. 2023 Jun 16;54:49. doi: 10.1186/s13567-023-01180-y (PMC10276399; doi:10.1186/s13567-023-01180-y)

Additional file 2. Fecal consistency scores for assessment of swine dysentery

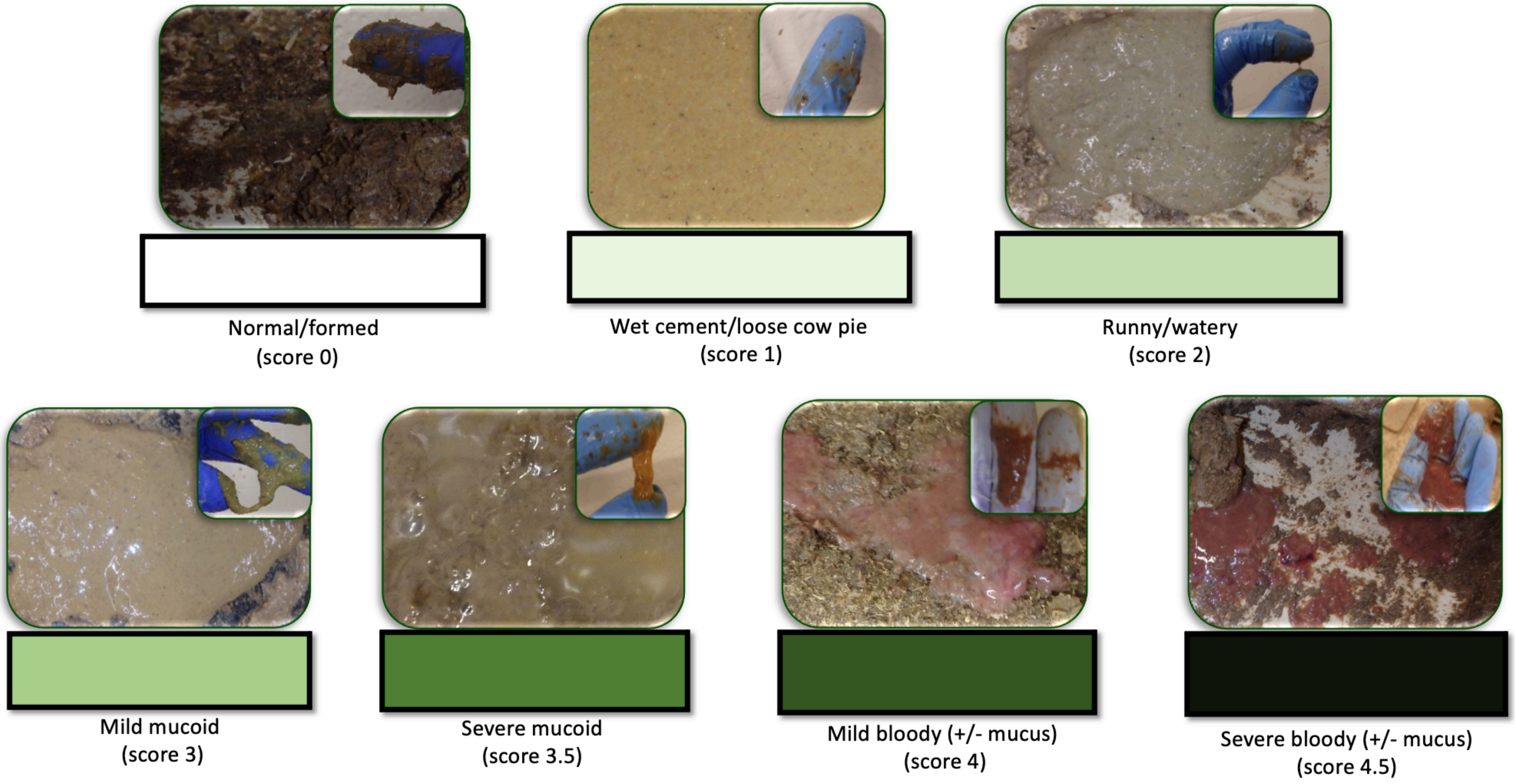

Supplement: Supplementary file 2 — Additional file 2: Fecal consistency score. [file 13567_2023_1180_MOESM2_ESM.pdf]
