# Supplementary material for: Improving the consistency of experimental swine dysentery inoculation strategies
Source: Vet Res. 2023 Jun 16;54:49. doi: 10.1186/s13567-023-01180-y (PMC10276399; doi:10.1186/s13567-023-01180-y)
Supplement: Supplementary file 3 — Additional file 3: Arrive 10 check list. [file 13567_2023_1180_MOESM3_ESM.pdf]

ARRIVE 10 guidelines 2.0

		Section/line number, or reason for not reporting
1. Study design	a. The groups being compared, including control groups. If no control group has been used, the rationale should be stated.	Experimental procedures
	b. The experimental unit (e.g. a single animal, litter, or cage of animals).	
2. Sample size	a. Specify the exact number of experimental units allocated to each group, and the total number in each experiment. Also indicate the total number of animals used.	
	b. Explain how the sample size was decided. Provide details of any a priori sample size calculation, if done.	The sample size was based on technical requirements of each trial (inoculum production) and availability of pig rooms. See Methods and Dicussion for more details.
3. Inclusion and exclusion criteria	a. Describe any criteria used for including and excluding animals (or experimental units) during the experiment, and data points during the analysis. Specity if these criteria were established a priori. If no criteria were set, state this explicitly.	Clinical assessment, termination, and necropsy exam were established a priori
	b. For each experimental group, report any animals, experimental units or data points not included in the analysis and explain why. If there were no exclusions, state so.	Result section. First paragraph of each trial
	c. For each analysis, report the exact value of n in each experimental group.	Experimental procedures
4. Randomisation	a. State whether randomisation was used to allocate experimental units to control and treatment groups. If done, provide the method used to generate the randomisation sequence.	
	b. Describe the strategy used to minimise potential confounders such as the order of treatments and measurements, or animal/cage location. If confounders were not controlled, state this explicitly.	
5.Blinding	Describe who was aware of the group allocation at the different stages of the experiment (during the allocation, the conduct of the experiment, the outcome assessment, and the data analysis).	
6. Outcome measures	a. Clearly define all outcome measures assessed (e.g. cell death, molecular markers, or behavioural changes).	Clinical assessment, termination, and necropsy exam
	b. For hypothesis-testing studies, specify the primary outcome measure, i.e. the outcome measure that was used to determine the sample size.	NA
7. Statistical methods	a. Provide details of the statistical methods used for each analysis, including software used.	Statistical analysis
	b. Describe any methods used to assess whether the data met the assumptions of the statistical approach, and what was done if the assumptions were not met.	
8.Experimental animals	a. Provide species-appropiate details of the animals used, including species, strain and substrain, sex, age or developmental stage, and, if relevant, weight.	Experimental procedures
	b. Provide further relevant information on the provenance of animals, health/immune status, genetic modification status, genotype, and any previous procedures.	
9. Experimental procedures	For each experimental group, including controls, describe the procedures in enough detail to allow others to replicate them, including:	
	a. What was done, how it was done and what was used.	General and laboratory procedures
	b. When and how often.	
	c. Where (including detail of any acclimatisation periods).	
	d. Why (provide rationale for procedures).	
10. Results	For each experiment conducted, including independent replications, report:	
	a. Summary/descriptive statistics for each experimental group, with a measure of variability where applicable (e.g. mean and SD, or median and range).	Table 3
	b. If applicable, the effect size with a confidence interval	NA
