# Supplementary material for: Improving the consistency of experimental swine dysentery inoculation strategies
Source: Vet Res. 2023 Jun 16;54:49. doi: 10.1186/s13567-023-01180-y (PMC10276399; doi:10.1186/s13567-023-01180-y)
Supplement: Supplementary file 4 — Additional file 4: Humane Intervention Point checklist. [file 13567_2023_1180_MOESM4_ESM.pdf]

Additional file 4. Humane Intervention Point (HIP) checklist

Clinical scoring system: Each inoculated pig will be observed twice daily (before 9 am and after 3 pm) and the clinical signs of disease assessed as follows: Note: If any clinical sign scores >= 2, medical intervention may be warranted.

SCORE	CLINICAL SIGNS
**A: RESPONSIVENESS**	
0	ALERT AND ACTIVE
1	ALERT, BUT SLOWER THAN PEN MATES
2	RELUCTANT TO MOVE, BUT GETS UP BY STIMULATION
3	DOWN, DOESN'T RESPOND WITH STIMULATION OR DEMONSTRATES SEIZURES (LEG PADDLING, RECUMBENCY, OPISTHOTONOUS) → *EUTHANASIA*
**B: COLOUR OF THE SKIN AND EXTREMITIES**	
0	NORMAL
1	SMALL AREA (< 50%) OF SUBCUTANEOUS HYPEREMIA ON THE EAR, TAIL AND ABDOMEN
2	> 50% OF THE EAR, TAIL OR ABDOMEN HAVE HYPEREMIA OR CYANOSIS, BUT NO OBVIOUS NECROSIS → *CONSIDER TREATMENT*
3	> 50% OF THE EAR, TAIL OR ABDOMEN HAVE HYPEREMIA OR CYANOSIS WITH NECROSIS OF THE SKIN → *EUTHANASIA*
**C: RESPIRATION**	
0	NORMAL
1	INCREASED RESPIRATION RATE
2	INCREASED RESPIRATION RATE, SLIGHT ABDOMINAL BREATHING (DYSPNEA) AND/OR COUGHING
3	INCREASED RESPIRATION RATE AND MARKED ABDOMINAL BREATHING (DYSPNEA) AND/OR PERSISTENT PAROXYSMAL COUGHING → *CONSIDER EUTHANASIA**
**D: CONSISTENCY OF THE FECES**	
0	FORMED, NORMAL
1	WET CEMENT OR LOOSE COW PIE
2	RUNNY OR WATERY
3	MUCOID
4	BLOODY OR REDDISH COLOURED (MAY OR MAY NOT BE MUCOID AS WELL) ⍰ MONITOR DURATION AND CONSIDER EUTHANASIA* IF >5 DAYS DURATION AND ACCOMPANIED WITH LOSS OF BODY CONDITION AND ROUGH HAIR COAT
**E: BODY CONDITION**	
0	NORMAL BODY CONDITION AND GUT FILL
1	NORMAL BODY CONDITION, HOLLOW FLANKS
2	SLIGHT LOSS OF BODY CONDITION, BACK BONE EVIDENT, WEIGHT LOSS UP TO 15% OF BODY WEIGHT
3	MODERATE LOSS OF BODY CONDITION, BACK BONE PROMINENT, WEIGHT LOSS GREATER THAN 15% OF
**F: BODY TEMPERATURE** – TAKEN ONLY IF SCORES IN A-E ARE >2	
0	37-39.5^{o}C
1	39.6-40.5^{o}C OR 36-36.9^{o}C
2	40.6-41.5^{o}C OR 35-35.9^{o}C
3	>41.5^{o}C OR <35°C → *CONSIDER EUTHANASIA**
